# B and T Immunoregulation: A New Insight of B Regulatory Lymphocytes in Autism Spectrum Disorder

**DOI:** 10.3389/fnins.2021.732611

**Published:** 2021-10-28

**Authors:** Andrea De Giacomo, Concetta Domenica Gargano, Marta Simone, Maria Giuseppina Petruzzelli, Chiara Pedaci, Donatella Giambersio, Lucia Margari, Maddalena Ruggieri

**Affiliations:** Department of Basic Medical Sciences, Neuroscience, and Sense Organs, University of Bari “Aldo Moro”, Bari, Italy

**Keywords:** ASD, autism, immune system, immune tolerance, regulatory T lymphocytes, regulatory B lymphocytes

## Abstract

**Introduction:** Autism Spectrum Disorder (ASD) is a heterogeneous neurodevelopmental disorder characterized by a complex pathogenesis, by impairment social communication and interaction, and may also manifest repetitive patterns of behavior. Many studies have recognized an alteration of the immune response as a major etiological component in ASDs. Despite this, it is still unclear the variation of the function of the immune response.

**Aim:** Our aim is to investigate the levels of immunological markers in peripheral blood of children with ASD such as: regulatory B and T cells, memory B and natural killer (NK) cells.

**Materials and Methods:** We assessed various subsets of immune cells in peripheral blood (regulatory B and T cells, B-cell memory and natural killer cells) by multi-parametric flow cytometric analysis in 26 ASD children compared to 16 healthy controls (HCs) who matched age and gender.

**Results:** No significant difference was observed between B-cell memory and NK cells in ASDs and HCs. Instead, regulatory B cells and T cells were decreased (*p* < 0.05) in ASD subjects when compared to HCs.

**Discussion:** Regulatory B and T cells have a strategic role in maintaining the immune homeostasis. Their functions have been associated with the development of multiple pathologies especially in autoimmune diseases. According to our study, the immunological imbalance of regulatory B and T cells may play a pivotal role in the evolution of the disease, as immune deficiencies could be related to the severity of the ongoing disorder.

## Introduction

Autism spectrum disorder (ASD) is a complex heterogeneous neurodevelopmental disorder characterized by impairments in social relationship and communication, manifesting repetitive and stereotyped behavior, and deficits in verbal and non-verbal interaction (APA, 2013).

ASD has an extensive clinical heterogeneity and it is correlated with numerous comorbidities including: social anxiety disorder, attention deficit disorder, immune system abnormalities, gastrointestinal disorders, mitochondrial dysfunction, sleep disturbances, and epilepsy (Mannion and Leader, 2013, 2016).

ASD is prominent for its complex disorder and uncertain pathogenesis. However, genetic, epigenetic, and environmental factors contribute to its development (Abrahams and Geschwind, 2008; Rossignol et al., 2014). Although the specific etiologies of ASD remain unknown, there are many hypotheses about the potential involvement of the immune system in the etiopathogenesis of the disease. Many studies have recognized an alteration of the immune response in individuals diagnosed with ASD. Evidence of alterations in the functioning of the central and peripheral immune systems reveal that there is a subset of individuals with ASD who have some form of immune dysregulation (Goines and Van de Water, 2010; Gottfried et al., 2015).

Besides, a high prevalence of other immune-related comorbidities including: autoimmune diseases, allergies, and psoriasis have been found in children with ASD compared to healthy controls (Zerbo et al., 2015). Alterations of the immune system include: improper stimulation of immune cells, generation of autoantibodies, cytokine/chemokine imbalance, and increased permeability of the blood-brain barrier (Gładysz et al., 2018). Individuals diagnosed with ASD often have an immunomodulation of the lymphocytes (T lymphocytes, B lymphocytes, monocytes, Natural Killer (NK) cells and dendritic cells).

Immune system aberrations, including altered cytokine profiles, are thought to associate with ASDs (Ashwood et al., 2006; Enstrom et al., 2009b; Goines and Van de Water, 2010; Jyonouchi, 2013; Gottfried et al., 2015; Masi et al., 2015; Mead and Ashwood, 2015; Gładysz et al., 2018; Hughes et al., 2018). Increased frequency of monocytes, myeloid dendritic cells (associated with bigger amygdala size and more aberrant behaviors), NK cells have been reported in children with ASD (Enstrom et al., 2009a; Ashwood et al., 2011; Breece et al., 2013).

Regulatory T and B lymphocytes play an important role in self-tolerance as they limit autoimmune responses by suppressing proinflammatory modes of action (Dasguptaa et al., 2020). It has been demonstrated that Tregs were decreased in some autoimmune disorders such as Multiple Sclerosis (MS), rheumatoid arthritis (RA) (Toubi et al., 2005) and clinically active systemic lupus erythematosus (LES) (Miyara et al., 2005). Alterations of Bregs instead have been found in the same diseases, in tumors and infectious diseases (Rincon-Arévalo et al., 2015). Variation in frequencies of T cells and in both mature and activated B cells have also been reported in ASDs. In particular, an altered function or a decrease in regulatory T lymphocytes (Tregs) was highlighted (Mostafa et al., 2010; Ashwood et al., 2011; Hughes et al., 2018). Tregs are essential for maintaining homeostasis of the immune system, limiting the extent of effector responses by allowing the creation of immunological tolerance.

Moreover, two main types of Tregs have been identified: natural Tregs and inducible ones. Natural Tregs are developed in the thymus compared to Inducible regulatory T (iTregs) which are “CD4^+^ lymphocytes” that express the transcription factor forkhead box P3 (Foxp3) and are developed outside the thymus under different conditions (Bilate and Lafaille, 2012). Recently, regulatory B lymphocytes (Bregs) have contributed to the maintenance of peripheral tolerance by limiting excessive inflammatory responses that occur during either autoimmune diseases or uncontrolled infections. Interleukin 10 (IL-10) is essential for Bregs function as it inhibits proinflammatory cytokines and supports the differentiation of Tregs (Mauri and Bosma, 2012).

It has been spotted that there is an increase in both T-helper 17 (Th17) lymphocytes (Th17) and cytokine Interleukin 17 (IL-17) in ASDs. A downregulation of Tregs and a decrease in Transforming Growth Factor beta (TGF-β) and Interleukin 10 (IL-10) production have also been detected. Therefore, a high “Th17” or “Treg ratio” has been attributed to severe ASD (Basheer et al., 2018; Moaaz et al., 2019). Reduced levels of the regulatory cytokine TGF-b1 are associated with reduced adaptive behavior and worsening behavioral symptoms (Ashwood et al., 2008; El Gohary et al., 2015).

To further underpin immunodeficiency disorder, Heuer et al. (2008) demonstrated that a reduction of IgM and IgG levels correlate with high behavioral severity in ASD patients. Therefore this review is designed to explore the alterations in various peripheral immune cell subpopulations and their roles in ASDs compare to healthy controls. The alteration of the immune regulation and inflammatory response are considered possible risk factors in the etiopathogenesis of ASD.

The main objective of our study is to focus on ASDs with Bregs and T regs alterations, and lack of immune tolerance. We hypothesized that a reduction of regulatory B and T cells could be related to the severity of the disease. Therefore, a better approach of the immune dysregulation and inflammation in ASDs might be essential in the diagnosis of this neurodevelopmental disorder.

## Materials and Methods

### Patients

Twenty-six patients (PTs) (mean age 8.3 ± 3.6 years) affected by ASD and 16 Healthy Controls (HCs) (mean age 9.9 ± 5.7 years) were enrolled for a follow—up at the Department of Child Neuropsychiatry, University of Bari, Italy (Table 1). The study-protocol was approved by the Local Ethics Committee and all patients agreed to the written informed consents. Hereafter, they were then diagnosed through clinical interviews with parents following DSM-5 criteria (APA, 2013) and through. Autism Diagnostic Observation Schedule (Lord et al., 2012). The ADOS provides an algorithm with cut-offs for autism and ASDs and four different modules were used, based on the language ability of the child.

**TABLE 1:** Demographic and clinical data.

Variable	ASD	HCs	*p*-value
Age; year (mean ± SD)	8.3 ± 3.6	9.9 ± 5.7	NS
Disorder duration; month (mean ± SD)	56.5 ± 35.9		
Gender, n (%)			
Male	21 (80.8)	12 (75.0)	NS
Female	5 (19.2)	4 (25.0)	NS

*SD, Standard deviation; n, sample size; %, percentage; NS, no statistically difference.*

Exclusion criteria in PTs are: the presence of genetic syndrome in particular Fragile-X syndrome, Rett’s syndrome and Tuberous Sclerosis, history of allergies, autoimmune disease, disabling neurological disease, pharmacological therapy, febrile seizure, and PTs receiving a special diet and with recent febrile illness.

Exclusion criteria in HCs are: the presence of neurodevelopmental disease, psychiatric or neurological disorders, pharmacological therapy, autoimmune diseases or allergic disorders.

Demographic and clinical data were collected from both ASD PTs and HCs: age, gender and disorder duration (period from the date of first diagnosis) (Table 1) and PTs were assessed for neurological disability which includes ASD level, IQ and language disability (Table 2). We opted for the *Wechsler Intelligence Scale for Children, fourth edition (WISC-IV)* to assess total/verbal IQ in scholar children (Wechsler, 2012); *Leiter International Performance Scale Revised-Visualization and Reasoning Battery (Leiter-R)* to assess not-verbal IQ in pre-scholar children (Roid and Miller, 2002); *Test di Valutazione del Linguaggio (TVL)* to evaluate language disability (Cianchetti, 2010).

**TABLE 2:** ASD level, IQ, and language disability.

Patients with severe ASD level, n (%)	6 (26%)
Patients with IQ < 70, n (%)	14 (54%)
Patients with language disability, n (%)	17 (65%)
Patients with full impairment, n (%)	5 (19%)

*n, sample size; %, percentage of patients with the mentioned characteristic among the whole group of patients.*

Hence, patients with greater impairments were examined according to four parameters: severe ASD level (severe: level 3; mild to moderate: level 1 and 2 according to DSM-5), intelligence quotient (IQ), language disability and the presence of all these parameters to identify patients with full impairments. The outcome result is as follows: 6 patients with severe ASD level, 14 patients with IQ < 70, 17 patients with language disability and 5 patients with full impairments.

### Flow Cytometric Analysis of Peripheral Lymphocytes Subsets

Blood samples were collected in fast blood specimens in ethylene diamine tetra acetic acid (EDTA) (8.55 mg/tube) for immunophenotyping by flow cytometry. Hundred microliter of peripheral blood samples were incubated with specific monoclonal antibodies (Beckman Coulter, United Kingdom) at room temperature (RT) for 15 min in the dark. After incubation, red cells were lysed with VersaLyse lysing solution (REF A09777, Beckman Coulter, United Kingdom) for 15 min at RT in the dark and then analyzed by flow cytometry. Analysis for samples incubated with Anti-FoxP3 was performed using PerFix-nc (no centrifuge assay Kit) (REF B31167, Beckman Coulter, United Kingdom), for intra- and extra-cellular staining preparation.

Lymphocytes subpopulations were identified by the recognition of surface molecules belonging to the family of Cluster of Differentiation (CD) and by intracellular FoxP3 transcription factor. Analyzed events were subjected to gating analysis (total lymphocytes and CD45^+^ vs. side scatter) (CD45-FITC, REF A07782, Beckman Coulter, United Kingdom) and exclusion of doublets and dead cells. Lymphocytes subpopulations were identified by using the monoclonal antibodies against CD3^+^/CD19^+^/CD27^+^ for B memory cells (CD3-PC5, REF A07749; CD19-PC7, REF IM3628; CD27-PE REF IM2578, Beckman Coulter, United Kingdom); CD19^+^/CD38^+^/CD24^+^ for B regulatory cells (CD19-PC7, REF IM3628; CD38-PE REF AO7779, CD24-PC5.5 REF B23133, Beckman Coulter, United Kingdom); CD4^+^/CD25^+^/CD127^+^/Anti-FoxP3^+^ for T regulatory cells (CD4-FITC, REF A07750; CD25-PE REF A07774, CD127-PC7 REF A64618, Anti-FoxP3-PC7 REF B46032, Beckman Coulter, United Kingdom); CD3^+^/CD16^+^/CD56^+^ for Natural Killer cells (CD3-PC5, REF A07749; CD16-PC7 REF 6607118; CD56-PE REF A07788, Beckman Coulter, United Kingdom). Samples were acquired on a Beckman Coulter CytoFLEX flow cytometer and analyzed using CytExpert software (Beckman Coulter, United Kingdom). Data are shown as the mean of percentage of events ± SD.

### Statistical Analysis

The lymphocytes’ subsets percentage in ASD patients and HCs one are reported as mean ± standard deviation (SD). Lymphocytes subsets values were not normally distributed, and therefore a Spearman non-parametric test was performed to compare these parameters with patients’ clinical and demographic data. Student’s *t*-test was used for statistical analysis on the percentage means of LS and Pearson Correlation coefficient was used for statistical analysis on participants’ gender. *P*-values < 0.05 were considered statistically significant. Thus, further analysis was conducted using GraphPad Prism 8.0.

## Results

### Lymphocytes Subsets

**FIGURE 1:**
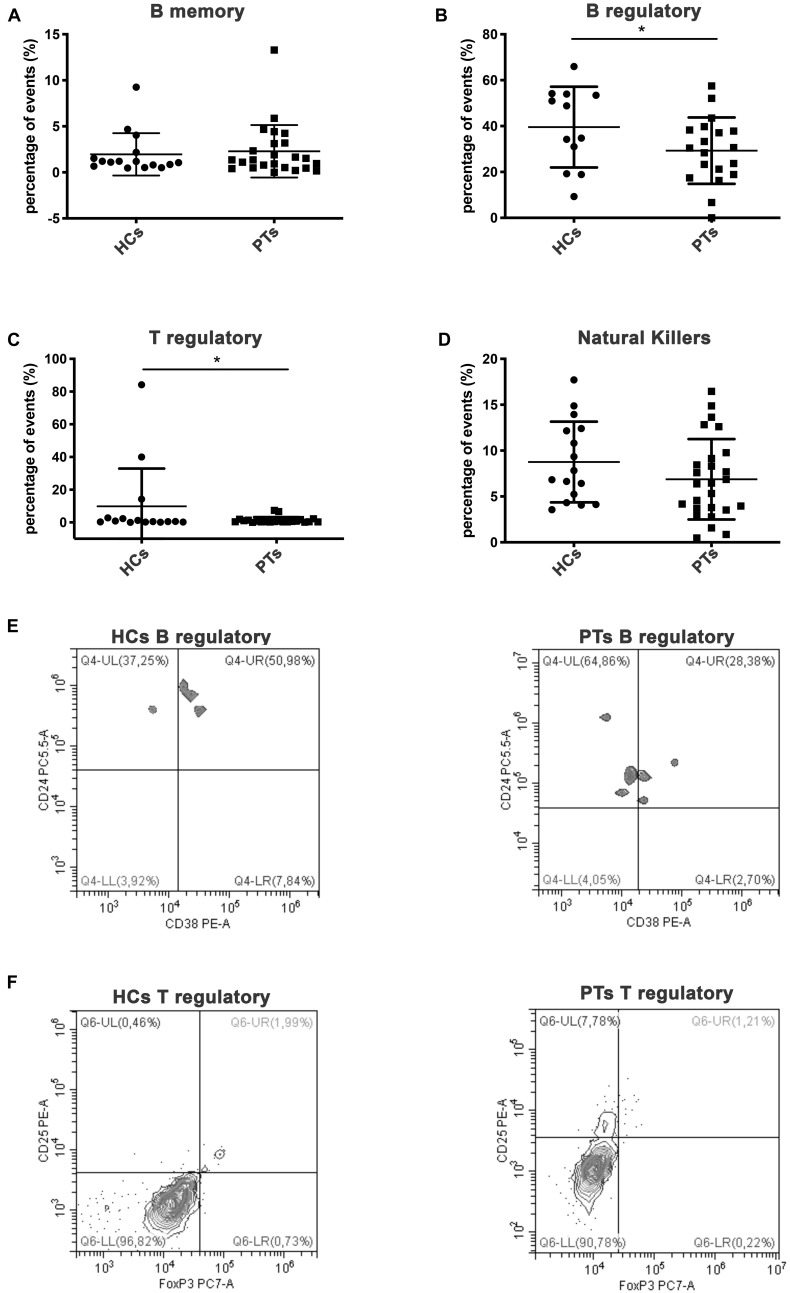
Lymphocytes subpopulations. Scatter plot graphs representing the percentage of events of lymphocyte subpopulations **(A)** memory B cells (CD45^+^/CD3^+^/CD19^+^/CD27^+^), **(B)** regulatory B cells (CD45^+^/CD19^+^/CD38^+^/CD24^+^), **(C)** regulatory T cells (CD4^+^/CD25^+^/FoxP3^+^), **(D)** natural killer (CD45^+^/CD3^+^/CD16^+^/CD56^+^); *p-value < 0.05 (Student’s *t*-test). Representative dot plot graphs of flow cytometry analysis of lymphocytes subpopulations. **(E)** B regulatory lymphocytes of HCs and PTs are shown in Q4-UR (underlined) representing CD38^+^/CD24 events previously gated on CD45^+^/CD19^+^. **(F)** T regulatory lymphocytes of HCs and PTs are shown in Q6-UR (underlined) representing CD25^+^/anti-FoxP3 events previously gated on CD4^+^.

Lymphocyte subsets (LS) cytometric analysis revealed some differences between patients (PTs) and HCs. Interestingly, statistical differences were shown for regulatory B cells subset (Figures 1B,E, Mean ± SD: HCs, 39.56 ± 17.6; PTs, 29.29 ± 14.5, *P* = 0.04) and for regulatory T cells subset (Figures 1C,F, Mean ± SD: HCs, 9.88 ± 23.1; PTs, 1.33 ± 1.9, *P* = 0.045) which were involved in immune tolerance mechanisms. Moreover, no statistical difference were found in B memory lymphocytes (Figure 1A, Mean% ± SD: HCs, 1.95 ± 2.3; PTs, 2.29 ± 2.9, *P* = 0.35) and NK cells (Figure 1D, Mean% ± SD: HCs, 8.76 ± 4.4; PTs, 6.87 ± 4.4, *P* = 0.09).

For a better survey of regulatory B and T lymphocytes subsets involvement in ASD, we compared the mean percentage of LS of patients with the most severe conditions to the mean percentage of LS of all patients. Table 3 identifies the mean percentage of regulatory B and T subsets in all PTs and in different subgroups of severity. The percentage of variation between these two lymphocytes population is shown in Table 4. Four out of six patients with severe ASD level showed a reduction of 35.3% in the T reg subset compared to the T reg subset of all patients (T reg mean% of level 3 vs. level 1, 2: 0.86 vs. 1.33). A reduction of 31.3% in the B reg subset was observed in patients with severe ASD level, compared to the B reg subset of all patients (B reg mean% of level 3 vs. level 1, 2: 20.11 vs. 29.29). Lymphocytes subsets are quite similar when compared to patients with IQ < 70 and with language disability to all the other patients. However, patients with full impairment showed a reduction of 26.3% in the T reg subset in comparison with children with mild to moderate autism (T reg mean% of level 3 vs. level 1, 2: 0.98 vs. 1.33) and a reduction of 31,3% in the B reg subset compared to all patients (B reg mean% of level 3 vs. level 1, 2: 20.11 vs. 29.29). No statistically differences were found comparing ASD patient groups on the basis of disease severity (*P*-value > 0.05; Student’s *t*-test).

**TABLE 3:** B and T regulatory subsets means: comparison between total PTs and sub- groups of severity.

Variable	Total PTs	Severe ASD	10 < 70	L. disability	Full impairment	*p*-value
B reg (mean%)	29.29	20.11	30.40	30.16	20.11	NS
T reg (mean%)	1.33	0.86	1.31	1.23	0.98	NS

*PTs, patients; L, disability: language disability; full impairment: PTs with severe ASD level, 10 < 70 and language disability; B reg, B regulatory subpopulation; T regulatory subpopulation; mean%, mean of lymphocyte subpopulation in percentage; NS, no statistically difference.*

**TABLE 4:** Percentage of variation vs. all patients.

	T reg	B reg	*p*-value
LS of PTs with severe ASD level, (%)	−35.3	−31.3	NS
LS of PTs with IQ < 70, (%)	−1.5	+3.8	NS
LS of PTs with language disability, (%)	−7.5	+2.9	NS
LS of PTs with full impairment, (%)	−26.3	−31.3	NS

*LS, Lymphocytes Subpopulation; PTs, patients; full impairment: PTs with severe ASD level, IQ < 70 and language disability; T reg, T regulatory subset; B reg, B regulatory subset; NS, no statistically difference.*

## Discussion

The alteration of regulatory T and B lymphocytes has been reported to be involved in ASD. It is difficult to make direct comparisons between results of our study and previous studies due to differences in analytical technique, age range of subjects, diagnostic criteria and the use of siblings as controls. In our case, children with autism had significantly lower frequency of both Tregs and B regs (*p* < 0.05) compared to HCs. On the contrary, there was no significant difference in the proportion of B memory and NK cells. Four out of six patients diagnosed with severe ASD (level 3, according to DSM-5) showed lower value of Tregs (reduction of 35.3%) compared to children with mild or moderate autism (level 1 and 2, according to DSM-5) (Tables 3, 4). However, the above results did not reach statistical significance.

According to our results, Mostafa et al. (2010) demonstrated a significantly lower frequency of Tregs in Egyptian autistic children compared to HCs. Children with severe autism had a major decrease in the frequency of Tregs in peripheral blood than children with mild and moderate autism (Mostafa et al., 2010). Another study found a dysregulation of Tregs related to transcription factors, in particular a downregulation of Foxp3 within CD4^+^ T cells in peripheral blood of autistic children. These results suggest that a reduction in Foxp3 expression is associated to a decrease of Tregs in children with autism (Ahmad et al., 2017). Moreover, Ahmad et al. also highlighted a systemic Treg deficiency, focusing on dysregulation of Th1, Th2, Th17, and Treg-related transcription factors. A deficiency of the Foxp3^+^ Tregs protein was also found in upregulation of Th1/Th2/Th17 related transcription factors. These findings suggest that transcription factor signaling is altered in ASDs, which results in immunological imbalance, and therefore, the restoration of the transcription factor signaling could have a greater therapeutic potential in the treatment of autistic disorders (Ahmad et al., 2017).

To validate our findings, there is also a study (Moaaz et al., 2019), demonstrating that Th17/Treg imbalance in ASDs was significantly skewed toward a Th17 response compared to their control. This means that there is a significant reduction in Treg percentage in ASDs and this also confirmed the downregulation of the related transcription factor (Foxp3) and cytokines (TGF-β and IL-10) in their peripheral blood. Moaaz et al. (2019) have also discovered a negative correlation between Tregs and disease severity, showing that children with severe autism have lower values of Tregs than children with mild or moderate autism.

Regulatory T cells function in children with autism showed a decrease of TGF-β1 levels (Ashwood et al., 2008) and a IL-10 production (Ashwood and Wakefield, 2006). Changes in social behaviors, for example, are associated with reduced TGFβ1 levels (Ashwood et al., 2008). Tregs reduction have been reported in subjects with allergic disorders such as asthma (Mamessier et al., 2008). The presence of comorbid allergic disorders could be a confounding factor in immunity studies in both individuals with ASD and the control group. However, neither subjects with ASD nor the HCs had such a comorbid allergic diagnosis. Disagreeing with our results, Basheer et al. (2018) found no significant differences between ASDs and the control groups in proportion of Tregs. Future studies could investigate the clinical effects of a therapy based on restoring the number of Tregs in the subgroup of ASD patients with Treg deficiency (June and Blazar, 2006). We found that Bregs decreased (*p* < 0.05) in subjects with ASD if compared to HCs as well. No other studies have highlighted these findings. A Breg reduction seems to be linked to worsen severity in autism. In fact, in our findings, four out of six patients with severe autism showed a drastic reduction of about 31.3% if compared to the B reg subset of all patients Bregs are also reduced (−31.3%) in PTs with full impairment compared to all PTs (Tables 3, 4).

Heuer et al. (2008) studied B cells functions and observed a decrease in the total levels of both IgG and IgM in peripheral blood of autistic children compared to typically developing controls (Heuer et al., 2008). They subsequently investigate whether the reduced plasma levels of IgG and IgM were the result of defective development, activation or function of B cells. Heuer et al. (2012) showed no differences in the number of B memory cells. This indicates that the decrease of Immunoglobulins (Igs) in autism is not the result of B cell dysfunction but it depends on the involvement of many immune cells (Heuer et al., 2012).

In agreement with our findings, Basheer et al. (2018) found no significant differences in the frequency of NK cells in subjects with ASD. They also found no significant alterations in frequencies of various subsets of B cells in ASDs compared to previous study, in which a higher number of mature, activated B cells was reported (Ashwood et al., 2011; Basheer et al., 2018). Contrarily to our results, previous studies have shown a significantly higher number of B and NK cells in children with autism than in controls (Enstrom et al., 2009a; Ashwood et al., 2011).

Some studies have demonstrated that autistic children undergoing Intravenous Immunoglobulins (IVIG) treatment have improvements in total aberrant behavior, irritability, hyperactivity, and social withdrawal and in some cases, the complete resolution of ASD symptoms. The therapeutic mechanisms of Intravenous Immunoglobulins (IVIG) are complex; they may provide therapeutic benefits for both autoimmune and inflammatory diseases through multiple different processes (Galeotti et al., 2017; Rossignol and Frye, 2021). Other agents instead could decrease antibodies, including MMF (immunosuppressive therapy), methotrexate, rituximab, and bortezomib. These therapies in ASDs are, however, controversial and under debate because of the limitations of the data from *in vitro* or animal-based studies, the high cost of the treatment and the undefined immunopathology of autism (Wong and White, 2016).

Some limitations are present in this study due to the small sample size, the reduced number of HCs compared to ASD subjects and the difference in frequency between males and females. The wide age range of participants too could be considered as another limitation because of the different impact on the immune cells value. Further studies are needed to scrutinize for one or more reliable biomarkers that will help elucidate the mechanism underlying the causes of autism and this could help to identify different subgroups within the autistic population designing effective treatments and redefining ASD on molecular, immunological, and biochemical background. Therefore, it is important to carry on with this research on Bregs which play an important role in immune tolerance and these data could be useful for future biomarkers and therapies in ASD subjects.

## Data Availability Statement

The raw data supporting the conclusions of this article will be made available by the authors, without undue reservation.

## Ethics Statement

The studies involving human participants were reviewed and approved by Comitato Etico Policlinico. Written informed consent to participate in this study was provided by the participants’ legal guardian/next of kin.

## Author Contributions

AD and MS: wrote the first draft of the manuscript and design a work. MR and CDG: substantial contributions to the conception or design of the work, the acquisition, and analysis and interpretation of data for the work. All authors contributed to the manuscript revision, read and approved the submitted version.

## Conflict of Interest

The authors declare that the research was conducted in the absence of any commercial or financial relationships that could be construed as a potential conflict of interest. The reviewer PI declared a shared affiliation, though no other collaboration, with the authors to the handling editor.

## Publisher’s Note

All claims expressed in this article are solely those of the authors and do not necessarily represent those of their affiliated organizations, or those of the publisher, the editors and the reviewers. Any product that may be evaluated in this article, or claim that may be made by its manufacturer, is not guaranteed or endorsed by the publisher.
